# FACTORS FACILITATING COLLABORATION AND ENGAGEMENT: BUILDING AND SUSTAINING STATEWIDE COALITIONS

**DOI:** 10.1093/geroni/igz038.2973

**Published:** 2019-11-08

**Authors:** Cristine B Henage, Ellen C Schneider, Ellen Roberts, Vicki Tilley, Jan Busby-Whitehead

**Affiliations:** 1 University of North Carolina, Chapel Hill, North Carolina, United States; 2 The University of North Carolina at Chapel Hill, Chapel Hill, North Carolina, United States; 3 The University of North Carolina at Chapel Hill School of Medicine, Chapel Hill, North Carolina, United States

## Abstract

Sustaining collaboration across multiple community-based organizations (CBOs) creates synergies and economies of scale to support age-friendly communities beyond the provision of direct services any single CBO can achieve. The Carolina Geriatrics Workforce Enhancement Program (CGWEP) created and sustained multiple statewide coalitions focused on geriatrics syndromes. More than 290 CBOs, including Area Health Education Centers, social services programs and nongovernmental organizations, meet quarterly to form linkages, promote education and build infrastructure to support rural and underserved older adults. Shared governance with pooled resources has been achieved because of a long history of partnership, mutually beneficial relationships, flexibility, and frequent communication. The strength of the partnership is evidenced by continued growth in number of CBOs, number of sponsored events, and number of referrals to CBOs. Two coalitions, focused on falls prevention and mental health respectively, have been adopted by partners and sustained beyond grant funding.

